# An X Chromosome Association Scan of the Norfolk Island Genetic Isolate Provides Evidence for a Novel Migraine Susceptibility Locus at Xq12

**DOI:** 10.1371/journal.pone.0037903

**Published:** 2012-05-29

**Authors:** Bridget H. Maher, Rod A. Lea, Miles Benton, Hannah C. Cox, Claire Bellis, Melanie Carless, Thomas D. Dyer, Joanne Curran, Jac C. Charlesworth, Julie E. Buring, Tobias Kurth, Daniel I. Chasman, Paul M. Ridker, Markus Schürks, John Blangero, Lyn R. Griffiths

**Affiliations:** 1 Genomics Research Centre, Griffith Health Institute, Griffith University, Queensland, Australia; 2 Department of Genetics, Texas Biomedical Research Institute, San Antonio, Texas, United States of America; 3 Menzies Research Institute Tasmania, Hobart, Tasmania, Australia; 4 Division of Preventive Medicine, Department of Medicine, Harvard Medical School, Brigham and Women’s Hospital, Boston, Massachusetts, United States of America; 5 Donald W. Reynolds Center for Cardiovascular Disease Prevention, Harvard Medical School, Brigham and Women’s Hospital, Boston, Massachusetts, United States of America; 6 Department of Neurology, University Hospital Essen, Essen, Germany; 7 INSERM Unit 708 - Neuroepidemiology, Paris, France; Emory University School Of Medicine, United States of America

## Abstract

Migraine is a common and debilitating neurovascular disorder with a complex envirogenomic aetiology. Numerous studies have demonstrated a preponderance of women affected with migraine and previous pedigree linkage studies in our laboratory have identified susceptibility loci on chromosome Xq24-Xq28. In this study we have used the genetic isolate of Norfolk Island to further analyse the X chromosome for migraine susceptibility loci.

An association approach was employed to analyse 14,124 SNPs spanning the entire X chromosome. Genotype data from 288 individuals comprising a large core-pedigree, of which 76 were affected with migraine, were analysed. Although no SNP reached chromosome-wide significance (empirical α = 1×10^−5^) ranking by P-value revealed two primary clusters of SNPs in the top 25. A 10 SNP cluster represents a novel migraine susceptibility locus at Xq12 whilst a 11 SNP cluster represents a previously identified migraine susceptibility locus at Xq27. The strongest association at Xq12 was seen for rs599958 (OR = 1.75, *P* = 8.92×10^−4^), whilst at Xq27 the strongest association was for rs6525667 (OR = 1.53, *P* = 1.65×10^−4^). Further analysis of SNPs at these loci was performed in 5,122 migraineurs from the Women’s Genome Health Study and provided additional evidence for association at the novel Xq12 locus (P<0.05).

Overall, this study provides evidence for a novel migraine susceptibility locus on Xq12. The strongest effect SNP (rs102834, joint P = 1.63×10^−5^) is located within the 5′UTR of the *HEPH* gene, which is involved in iron homeostasis in the brain and may represent a novel pathway for involvement in migraine pathogenesis.

## Introduction

Migraine is a complex neurovascular disorder that affects an estimated 18% of adult women and 6% of adult men [1], [2]. An individual’s risk of suffering migraine depends on a complex interplay of genetic and environmental factors. Numerous triggers have been identified that may influence the onset of a migraine, however the exact underlying molecular mechanisms are still unknown.

Inheritance studies have shown migraine to run in families with heritability estimates ranging between 0.34–0.57 [3], [4] and numerous susceptibility loci have been identified using linkage and association studies. However, the complex nature of the disorder and the heterogeneity of the phenotypic manifestation have made it difficult to identify shared genetic components in outbred population studies. The use of genetic isolates may be useful for mapping migraine genes because of the reduced genetic and environmental diversity.

The Norfolk Island population is a genetic isolate that is descendant from 11 British ‘Bounty’ mutineers and 6 Tahitian women and family histories can be traced through detailed genealogical databases to the original founders. Geographical isolation as well as strict quarantine and immigration laws have also resulted in a more homogenous lifestyle then are seen in an outbred population [5]. Overall it is expected that the genetic diversity is reduced consequently simplifying genetic models and minimising environmental effects synergistically increasing the possibility of identifying susceptibility genes [6].

The prevalence of migraine in the Norfolk Island population is estimated at 25.5%, which is substantially higher then is seen in outbred populations. The heritability of the disorder is also substantial (H^2^ = 0.54) and consistent with other populations [7]. The prevalence of migraine in the Norfolk Island population also follows a similar epidemiological trend with women affected ∼3∶1 over men. This increased prevalence in women may be partially attributed to genes located on the X chromosome. Previous work from our laboratory has implicated X chromosome loci at Xq24, Xq27 and Xq28 in Australian migraine pedigrees [8], [9], [10].

Here we expand on the notion of X linked migraine genes by scanning the X chromosome in the high risk Norfolk Island isolate using a novel pedigree-based association approach.

## Results

To investigate the possible involvement of an X chromosome susceptibility gene in migraine we conducted a chromosome wide SNP association analysis in a very large Norfolk Island pedigree consisting of 288 individuals including 76 migraine cases (22 male, 54 female). From the 15,668 SNPs genotyped 14,124 were used for analysis, with the remainder discarded due to low genotyping call rates or low minor allele frequency.

### Single Marker Analysis

**Figure 1:**
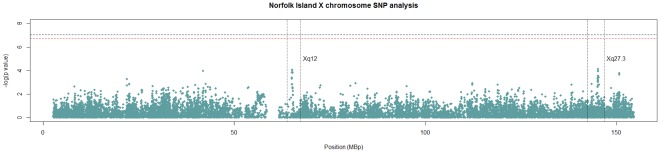
Norfolk Island X chromosome Scan.

A first pass X chromosome scan was undertaken using a logistic regression analysis that adjusted for age and gender. This approach did not incorporate any adjustment for kinship/relatedness in the cohort to allow for a computationally rapid first pass assessment of SNP rankings. A Manhattan plot of *P*-values from this analysis is depicted in Figure 1. The plot clearly shows three tightly defined peaks at the previously implicated loci Xq27 and Xq28 as well as a new locus on Xq12.

The SNPs were then prioritised according to the approximate P value and the top 25 SNPs were re-analysed in SOLAR using a linkage-based probit regression model to account exactly for the relatedness of the pedigree cohort. The 25 top ranked SNPs (see Table 1) clearly show a cluster of SNPs at the previously linked loci at Xq27 and Xq28. 11 SNPs localised to the previously identified loci on Xq27 (strongest association at rs6525667 (*P* = 1.65×10^−4^) and Xq28 (strongest association at rs6627483 *P* = 3.87×10^−3^). Interestingly 10 of the 25 top ranked SNPs also mapped to a new 377 Kb locus at Xq12, with the strongest association seen at rs599958 (*P* = 8.92×10^−4^) in this novel implicated region.

**Table 1:** Top 25 SNPs ranked by P-value.

Locus	SNP	MAF		OR	95% CI		P-value
		cases	controls		Lower	Upper	
Xq27	rs6525667	0.28	0.47	1.53	1.23	1.91	1.65×10−4
Xp11	rs5918294	0.55	0.36	1.48	1.20	1.81	1.67×10−4
Xq27	rs910618	0.29	0.48	1.49	1.19	1.86	3.67×10−4
Xq27	rs1998005	0.32	0.53	1.43	1.15	1.77	6.13×10−4
Xq27	rs1339482	0.35	0.54	1.42	0.18	11.57	7.39×10−4
Xq12	rs599958	0.16	0.05	1.75	1.21	2.53	8.92×10−4
Xq27	rs12555969	0.32	0.50	1.42	1.16	1.74	1.00×10−3
Xq12	rs5918577	0.16	0.06	1.73	1.24	2.40	1.13×10−3
Xq27	rs5920067	0.32	0.50	1.41	1.14	1.74	1.13×10−3
Xq12	rs760867	0.16	0.06	1.68	1.23	2.29	1.63×10−3
Xq27	rs12014291	0.37	0.53	1.4	1.13	1.73	1.69×10−3
Xq12	rs670546	0.16	0.05	1.71	1.27	2.29	1.34×10−3
Xq12	rs1028348	0.15	0.05	1.71	1.22	2.41	2.33×10−3
Xq12	rs5965083	0.16	0.06	1.65	1.19	2.28	2.41×10−3
Xq12	rs5918974	0.16	0.05	1.64	1.19	2.26	2.48×10−3
Xq12	rs5964480	0.16	0.06	1.62	1.18	2.23	3.09×10−3
Xq27	rs5920061	0.37	0.52	1.37	1.10	1.70	3.23×10−3
Xq28	rs6627483	0.25	0.10	1.47	1.13	1.91	3.87×10−3
Xq12	rs6525038	0.16	0.06	1.58	1.18	2.13	4.10×10−3
Xq12	rs6525037	0.16	0.06	1.57	1.15	2.15	4.87×10−3
Xq27	rs5920070	0.34	0.53	1.43	1.16	1.78	5.65×10−3
Xq27	rs5919666	0.55	0.38	1.32	1.07	1.63	8.00×10−3
Xq27	rs5920197	0.38	0.21	1.35	1.08	1.68	8.14×10−3
Xq28	rs12843815	0.52	0.31	1.31	1.07	1.61	8.87×10−3
Xp22	rs2071201	0.20	0.09	1.38	1.05	1.81	2.12×10−2

### Follow-up (Validation) Analysis

The Xq12, Xq27 and Xq28 loci were assessed in the independent Women’s Genome Health Study (WGHS) cohort. Thirty-one SNPs across the three loci were analysed consisting of 21 of the top 25 prioritised SNPs in the Norfolk population and 10 additional SNPs that ranked within the top 100 that also map to these loci. In these regions evidence of validation was observed for the Xq12 locus at three SNPs with the strongest association observed at rs1028348 (OR = 4.479 *P* = 7×10^−3^), which is located within the 5′UTR of the hephaestin (*HEPH*) gene. No association was observed at markers analysed in the Xq27 or Xq28 loci (Table 2) in the tested WGHS cohort.

### Haplotype Analysis

**Figure 2:**
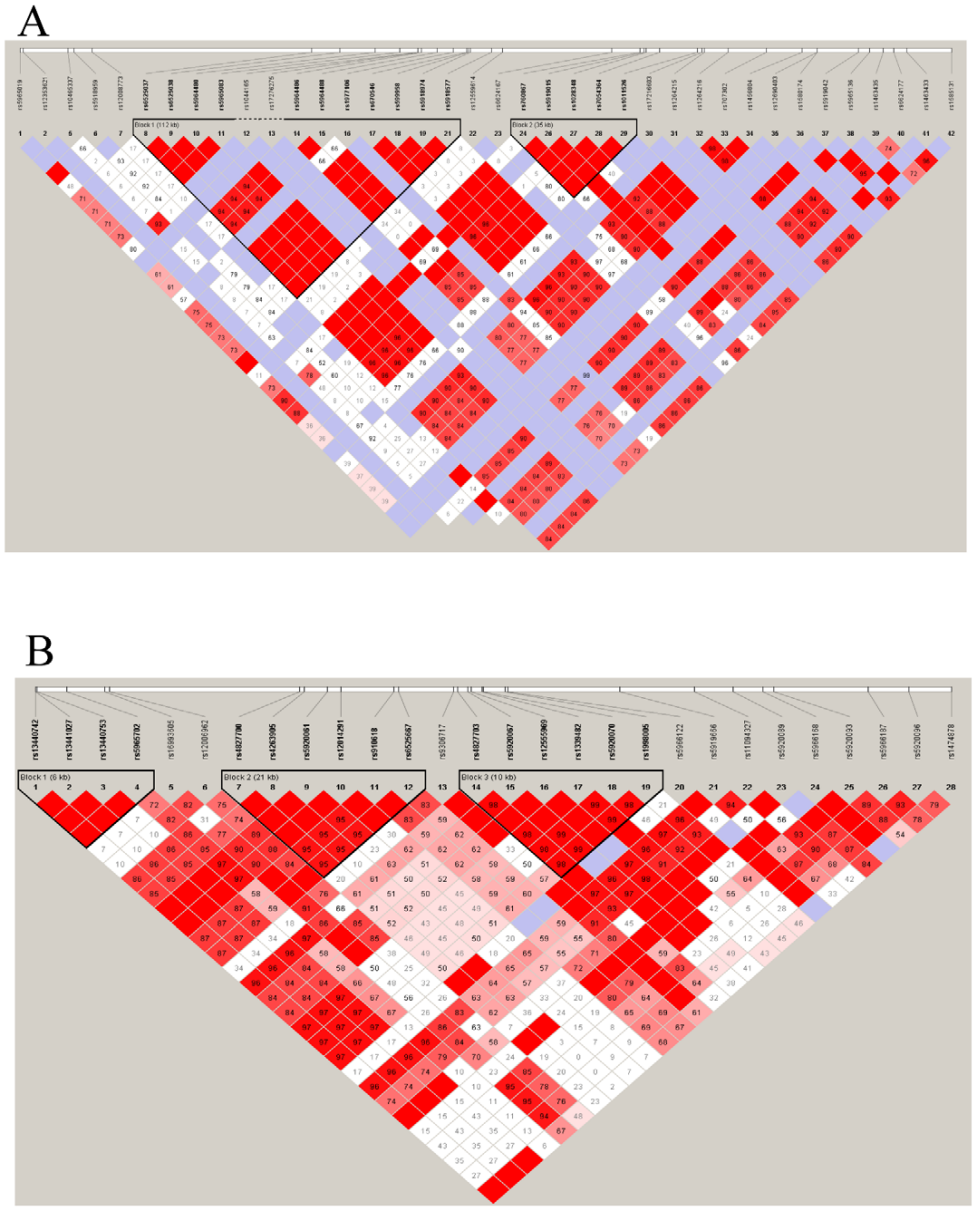
Linkage Disequilibrium plots. a) Xq12 locus b) Xq27 locus.

The novel Xq12 SNP cluster was considered for further haplotype analysis. The 377 kb Xq12 locus included 21 SNPs incorporating 10 of the top 25 ranked SNPs from the study. A linkage disequilibrium plot of the region was generated in Haploview and 2 major haplotype blocks were identified (Figure 2a). Block 1 consisted of 11 analysed SNPs. Analysis of this block revealed 3 haplotypes with haplotype 2 significantly over-represented in migraineurs (OR = 4.39 *P* = 1.1×10^−4^). Similarly block 2 included 5 genotyped SNPs representing 5 haplotypes. Of these, haplotype 1 was significantly over-represented in migraineurs (OR = 4.48 *P* = 1.6×10^−4^). The effect of this haplotype block appears to be due to the presence of the minor allele at rs1028348, located within the 5′UTR of the *HEPH* gene. Table 3 includes haplotype and frequency data for this locus.

The 2^nd^ SNP cluster identified includes 10 the top 25 ranked SNPs that localise to a previously identified Xq27 migraine locus. Haplotype analysis in the Xq27 region revealed 2 haplotype blocks (Figure 2b). Block 1 consisted of 6 SNPS and 5 haplotypes were identified. Of these haplotype 1 was significantly underrepresented in migraineurs (OR = 2.63 *P* = 1.3×10^−4^), while haplotype 5 was significantly overrepresented (OR = 2.3 *P* = 4×10^−4^). Similarly the second block also consisted of 6 SNPs with 4 different haplotypes recognised. Haplotype 1 was again underrepresented in cases (OR = 2.22 *P* = 6.9×10^−4^) and haplotype 3 significantly overrepresented in migraineurs (OR = 2.06 *P* = 1.6×10^−3^). Table 4 includes haplotype and frequency data for this locus.

For this study we decided not to pursue haplotypic follow-up in the WGHS because of the substantially different LD structure likely to exist between NI and WGHS cohorts as well as the differences in SNP typed.

## Discussion

The female preponderance of migraine suggests the disease is partly influenced by X-linked genomic factors. To date migraine susceptibility loci have been convincingly mapped to Xq24–28 and suggestively mapped to Xp22, mainly via pedigree linkage studies [8], [9], [10], [11]. For complex disease such as migraine the interplay between multiple genetic factors can make it difficult to distinguish the role each factor plays in the pathophysiology of disease. In an effort to address this, the genetic isolate of Norfolk Island was used to conduct a pedigree-based association study of the X chromosome. This population was used due to its high prevalence of migraine [7] and its inimitable pedigree structure in which genetic relationships can be traced through genealogical data to 17 original founders. The founder effect history of this population has led to a decrease in genetic variation which, when combined with homogeneity of environmental influences may improve the likelihood of detecting genetic haplotypes associated with migraine.

We analysed the X chromosomal data from a recent genome-wide association study [7] using a two-step method. We decided to separate the X chromosome from the autosomal analysis due to the specific X-linked hypothesis being tested and our previous findings regarding X chromosome loci and migraine [8], [9], [10]. Step one involved a preliminary scan of the data using a logistic regression association study approach controlling for age and gender. This preliminary step was a first pass screen carried out for computational efficiency due to a lack of appropriate software that could deal with the complexity of the pedigree structure, combined with the hemizygote nature of X chromosome data in males as well as automating the analysis of 14,124 SNPs. Step 1 was limited to estimation of the chromosome-wide profile and relative strength of SNP association. Step two involved a more intensive analysis of a vastly reduced number of SNPs using the program SOLAR [12] to exactly account for the relatedness of the pedigree. Using this approach we observed the same associations at the previously identified regions on Xq27 and Xq28. Single marker analysis also revealed a cluster of associated SNPs at a novel Xq12 locus and most of these SNPs showed evidence of validation in a large general migraine cohort. A SNP at Xp22 (rs2071201) also showed nominal evidence of association, which provides some support for a previously reported linkage peak at this locus. Overall, the magnitude of the associations seen in the Norfolk pedigree were greater than that observed in the general migraine population which adds support to the Norfolk isolate being a useful resource for disease gene discovery and functional studies.

**Table 2:** WGHS follow-up analysis.

		MAF		Migraine			
Locus	SNP	Cases	Controls	OR	L95CI	U95CI	*P*
**Xq12**	rs6525037	0.102	0.106	1.04	0.97	1.12	0.24
	rs6525038	0.096	0.100	1.05	0.97	1.13	0.23
	rs5964480	0.099	0.103	1.05	0.98	1.13	0.19
	rs5965083	0.130	0.135	1.05	0.98	1.12	0.15
	rs5964486	0.150	0.158	1.06	1.00	1.13	0.068
	rs5964488	0.160	0.167	1.06	1.00	1.13	**0.049**
	rs670546	0.132	0.138	1.06	1.00	1.14	0.063
	rs5918974	0.132	0.138	1.06	1.00	1.14	0.062
	rs760867	0.132	0.139	1.07	1.00	1.14	**0.047**
	rs1028348	0.106	0.116	1.10	1.03	1.18	**0.007**
	rs7054364	0.212	0.221	1.05	0.99	1.11	0.076
	rs1011526	0.212	0.221	1.05	0.99	1.11	0.077
	rs1264216	0.214	0.221	1.04	0.99	1.10	0.12
**Xq27**	rs4827700	0.417	0.411	1.02	0.98	1.07	0.27
	rs4263905	0.417	0.411	1.02	0.98	1.07	0.26
	rs5920061	0.417	0.411	1.02	0.98	1.07	0.28
	rs12014291	0.451	0.445	1.03	0.98	1.07	0.25
	rs910618	0.375	0.373	1.01	0.97	1.06	0.61
	rs6525667	0.375	0.373	1.01	0.97	1.06	0.62
	rs4827703	0.498	0.493	1.02	0.97	1.06	0.42
	rs5920067	0.423	0.420	1.01	0.96	1.05	0.65
	rs12555969	0.381	0.379	1.01	0.96	1.05	0.79
	rs1339482	0.440	0.436	1.01	0.97	1.06	0.57
	rs5920070	0.441	0.437	1.01	0.97	1.06	0.54
	rs1998005	0.441	0.437	1.01	0.97	1.06	0.52
	rs5919666	0.486	0.91	1.02	0.98	1.07	0.35
	rs5920197	0.405	0.401	1.01	0.97	1.06	0.58
	rs11094364	0.211	0.209	1.01	0.96	1.07	0.63
**Xq28**	rs5970126	0.108	0.107	1.01	0.94	1.08	0.72
	rs12843815	0.455	0.453	1.01	0.97	1.05	0.65
	rs6627483	0.125	0.122	1.07	0.96	1.20	0.21

* Analysis of the MA subtype showed no statistically significant results, U95CI = upper 95^%^ confidence interval.

It is well known that the common forms of migraine are influenced by multiple small to moderate effect loci. Recent GWAS analyses were able to detect some of these loci as statistically significant at the genome-wide level, but only with much larger sample sizes [13]. For our study of the Norfolk Island pedigree the largest effect size was OR = 1.75 (*P* = 8.92×10^−4^). This statistic, whilst suggestive of association with migraine, fell slightly short of meeting our empirical threshold of statistical significance for the X chromosome (1×10^−5^). Failure to achieve greater effect sizes and statistical significance probably reflects the complex genetics of migraine and the limited size of the Norfolk population.

**Table 3:** Xq12 Haplotype Analysis.

HAPLOTYPE	Frequency				
Block 1*	Cases	Controls	OR	T statistic (Wald Test)	*P*
GAGGTTCCCTC	0.76	0.85	1.47	1.89	0.16
TGTACCCTACA	0.17	0.05	4.39	15.0	**1.1**×**10^−4^**
GAGGTTTCCTC	0.07	0.10	1.61	1.29	0.25
**Block 2** +					
GCAAT	0.15	0.05	4.48	14.2	**1.6**×**10^−4^**
GCGAT	0.02	0.01	1.67	0.27	0.601
ACGAT	0.06	0.06	1.01	4.4×10^−4^	0.983
ATGCC	0.24	0.26	1.14	0.31	0.574
ACGCC	0.53	0.62	1.40	2.50	0.114

* Block 1 consists of SNPs: rs6525037 rs6525038 rs5964480 rs5965083 rs5964486 rs5964488 rs1977106 rs670546 rs599958 rs5918974 rs5918577.

+ Block 2 consists of SNPs: rs760867 rs5919015 rs1028348 rs7054364 rs1011526.

The 377 kb Xq12 locus identified by SNP prioritization contains 2 genes, hephaestin (*HEPH*) and V-set and immunoglobin domain containing 4 (*VSIG4*) as well as 4 pseudogenes and 1 microRNA - *mir223. VSIG4* is coded in the middle of the first LD region identified in our haplotype analysis. This gene is part of the complement receptor family and appears to play an important role in regulating innate and adaptive immune response through clearance of autologous and pathogenic cells [14].

Similarly the 5′ end of *HEPH* gene is coded in the 2^nd^ LD block. While none of the tested SNPs are exonic, 1 SNP rs1028348 (*P* = 2.33×10^−3^ in the Norfolk study, *P* = 7×10^−3^ in the WGHS), is in the 5′UTR of the *HEPH* gene. Further sequencing of this locus is required to identify any additional SNPs that may be relevant to migraine. Hephaestin is an iron transport protein involved in cellular iron export through oxidising ferrous to ferric iron for uptake by transferrin or other iron carriers. Hephaestin expression has been identified throughout the human gastrointestinal tract as well as in pancreatic islets and the enteric nerves [15]. Qian and colleagues [16] also determined that this protein is expressed in the cortex, hippocampus, striatum and subtrantia nigra of rats and that development and iron status have a significant effect on the expression of the *HEPH* gene. Furthermore, it has been shown in mice that hephaestin is required for iron homeostasis in the CNS [17].

We have provided compelling evidence for a new migraine susceptibility locus at Xq12 in a pedigree from the genetic isolate of Norfolk Island and shown some evidence that the association extends to the general migraine population. Furthermore analysis in the Norfolk Island cohort provides additional evidence to support an existing migraine locus at Xq27.

**Table 4:** Xq27 Haplotype Analysis.

HAPLOTYPE	Frequency				
Block 1*	Cases	Controls	OR	T statistic (Wald Test)	*P*
AGTCGA	0.29	0.47	**2.63**	14.5	**1.3**×**10^−4^**
AGTCAG	0.07	0.03	2.42	3.06	0.080
GGTCAG	0.007	0.013	1.40	0.09	0.77
GTTCAG	–	0.02	NA	NA	NA
GTCTAG	0.63	0.47	2.30	12.30	**4.6**×**10^−4^**
**Block 2** +					
TCTGTG	0.33	0.51	2.22	11.5	**6.9**×**10^−4^**
TTCGTG	–	0.02	NA	NA	NA
CTCAGA	0.57	0.40	2.06	9.93	**1.6**×**10^−3^**
TTCAGA	0.10	0.07	1.54	1.15	0.283

* Block 1 consists of SNPs: rs4827700 rs4263905 rs5920061 rs12014291 rs910618 rs6525667.

+ Block 2 consists of SNPs: rs4827703 rs5920067 rs12555969 rs1339482 rs5920070 rs1998005.

## Methods

### Sample Ascertainment

The study protocol was approved by the Griffith University Human Research Ethics Committee and all participants provided signed informed consent. 600 participants were included based on permanent resident status to ensure sampling from a shared genealogical background. A comprehensive medical questionnaire was used to obtain phenotypic data including migraine information regarding family history, symptoms, triggers and medication. Migraineurs were diagnosed in accordance with ICHD-II guidelines [18].

Venous blood samples were obtained from the 600 participants (261 males, 339 females) with a mean age of 50.8 years (standard deviation 16.4 years). Blood samples were collected in EDTA tubes and DNA extracted from 10–20 mls using a standard salting out procedure [19]. Concentration and purity of the DNA yields were determined spectrophotometrically using the NanoDrop ND-1000 (NanoDrop Technologies, Inc.).

### Population

Genealogical data was obtained via questionnaire, and municipal and historical records. These records indicate Pitcairn Island was settled by 9 Isle of man ‘Bounty’ mutineers, 12 Tahitian women and 6 Tahitian men in 1790 [5]. Pedigree reconstruction and validation has confirmed current descendents possess lineages to all 9 ‘Bounty’ mutineers, 6 of the Tahitian women and 2 additional Caucasian sailors who joined the small colony during the early 19^th^ century [20], [21], [22]. A total of 377 individuals were determined to have familial links to these 17 founders and were integrated into heritability analyses. The size and complexity of the genealogical structure (N = 6,537) and large volume of missing data prohibited direct use in variance component linkage analysis [6]. To facilitate analysis, the pedigree was split (*N* = 1,078) using a peeling algorithm in the pedigree database management system PEDSYS. This 1,078 member pedigree has been previously employed in genome-wide screens of cardiovascular risk traits [6].

The mode of inheritance of migraine in this extended pedigree does not follow a clear Mendelian pattern ie. is complex. However, there is only a single observable case of male-to-male transmission which is consistent with a predominantly X chromosomal inheritance of migraine in this pedigree.

### Genotyping

A subset of 288 related individuals consisting of 136 males and 152 females were genotyped for this study including 76 migraine cases consisting of 22 males and 54 females. 15,668 X chromosome wide SNPs were genotyped on the Illumina Infinium High Density (HD) Human 610-Quad DNA analysis BeadChip version 1 using 200 ng of DNA per sample, as part of a GWAS. Samples were scanned on the Illumina BeadArray 500GX Reader and the Illumina BeadScan image data acquisition software (version 2.3.0.13) was used to collect raw data. Preliminary analysis of raw data was undertaken in Illumina GenomeStudio software (V2010.1) with the recommended parameters for the Infinium assay. Genotype cluster files were generated based on clustering of genotypes within the Norfolk Island population. Five individuals had a call rate below 95% and were not included in the analysis. SNPs were excluded from the analysis if a) the call rate fell below 99% b) genotype frequencies deviating from Hardy-Weinberg Equilibrium (P_HWE_<1×10^−5^) c) the a minor allele frequency (MAF) was less than 1% d) there were male heterozygosity scores observed [23].

### Statistical Analysis

For computational efficiency a logistic regression approach implemented in the PLINK software [23] was employed as a preliminary analysis for SNP association. Migraine affection status was set as a binary outcome variable and additive effects of SNPs were calculated after adjusting for covariates age and gender. A total of 14,124 SNPs spanning the X chromosome were analysed. We estimated the chromosome-wide statistical significance threshold based on empirical calculations performed by the simpleM program. Considering inter-SNP dependence across the chromosome we estimated the number of effective tests to be ∼4000 and thus set the significance threshold to 1×10^−5^ [24]. SNP results were annotated using the Whole Genome Association Study Viewer (WGAViewer) program [25] and NCBI build 37.1. A secondary analysis was also performed on the top 25 SNPs prioritised by *P* value. This analysis was undertaken using polygenic analysis in the SOLAR (Sequential Oligogenic Linkage Analysis Routines) software package [12]. This analysis uses a variance components-based linkage model to determine the polygenic heritability and the proportion of variance caused by the covariates, therefore handling the pedigree structure of the cohort in an exact fashion. SNPs were coded 0, 1, 2 for genotypes AA AB BB in females respectively and 0, 2 for genotypes A, B in males.

Haplotype analysis was carried out firstly using Haploview [26] to identify regions of linkage disequilibrium across key loci. Haplotype based association was then determined using PLINK to obtain haplotype frequencies and associations while controlling for age, gender and relatedness.

### Follow-up (Validation) Population

SNPs identified in the haplotype blocks at the Xq12 and Xq27 locus as well as significant SNPs in the Xq28 locus were investigated in an independent follow-up cohort from the Women’s Genome Health Study (WGHS). All SNPs that were present in the top 25 SNPs ranked by *P*-value were carried through for assessment in the follow-up cohort from the WGHS. Ascertainment and ethical approval of this cohort are described elsewhere [27]. The WGHS is derived from participants of the Women’s Health Study (WHS) and consists of 23,294 unrelated women of European descent. A total of 5122 women reported migraine during the study [13]. Statistical analysis methods used for the follow-up analysis of the WGHS are described in detail elsewhere [13].
